# Risk of endometriosis progression in infertile women trying to conceive naturally or using IVF

**DOI:** 10.1093/humrep/deaf090

**Published:** 2025-05-09

**Authors:** Edgardo Somigliana, Paola Vigano’, Dalila Invernici, Gianfranco Fornelli, Camilla Erminia Maria Merli, Paolo Vercellini

**Affiliations:** Department of Clinical Sciences and Community Health, Academic Center for Research on Adenomyosis and Endometriosis, Università degli Studi di Milano, Milan, Italy; Fondazione IRCCS Ca’ Granda Ospedale Maggiore Policlinico, Milan, Italy; Fondazione IRCCS Ca’ Granda Ospedale Maggiore Policlinico, Milan, Italy; Department of Clinical Sciences and Community Health, Academic Center for Research on Adenomyosis and Endometriosis, Università degli Studi di Milano, Milan, Italy; Fondazione IRCCS Ca’ Granda Ospedale Maggiore Policlinico, Milan, Italy; Department of Clinical Sciences and Community Health, Academic Center for Research on Adenomyosis and Endometriosis, Università degli Studi di Milano, Milan, Italy; Fondazione IRCCS Ca’ Granda Ospedale Maggiore Policlinico, Milan, Italy; Department of Clinical Sciences and Community Health, Academic Center for Research on Adenomyosis and Endometriosis, Università degli Studi di Milano, Milan, Italy; Department of Clinical Sciences and Community Health, Academic Center for Research on Adenomyosis and Endometriosis, Università degli Studi di Milano, Milan, Italy; Fondazione IRCCS Ca’ Granda Ospedale Maggiore Policlinico, Milan, Italy

**Keywords:** endometriosis, endometrioma, IVF, ART, recurrence, infertility, pregnancy

## Abstract

The use of hormonal treatments for endometriosis has increased in recent years. Their effectiveness lies in creating a stable hormonal environment, reducing peripheral estrogen levels, and suppressing ovulation and menstruation. Although these agents do not cure endometriosis and symptoms often return after discontinuation, they effectively relieve pain in most cases and help prevent disease progression or recurrence. Women are therefore typically managed with long-term hormonal treatments, with or without surgery. However, this approach is unsuitable for those seeking natural pregnancy or undergoing IVF, as all hormonal treatments hinder conception. For women pursuing natural pregnancy, these treatments should be discontinued for about 1 year, the time needed to diagnose infertility. However, this suspension exposes women to the risk of recurrence or progression and is therefore clinically acceptable only if the woman has a reasonable likelihood of achieving pregnancy naturally. In women with endometriosis who are infertile and therefore require IVF, ovarian stimulation significantly raises estrogen levels—up to 10 times those of a natural cycle—potentially boosting the risk of endometriosis relapse. Evidence is reassuring on this issue even if some limited data suggest that ovarian stimulation may promote deep invasive endometriosis progression. Overall, physicians and patients must balance the chances of natural or ART-assisted pregnancy against the risk of disease recurrence or progression during treatment discontinuation or IVF. This choice is also complicated by the increased risk of severe pregnancy complications in women with endometriosis, possibly depending on the conception method. This review discusses the available evidence that can be helpful in guiding the decision-making process.

## Graphical abstract

**Graphical Abstract:**
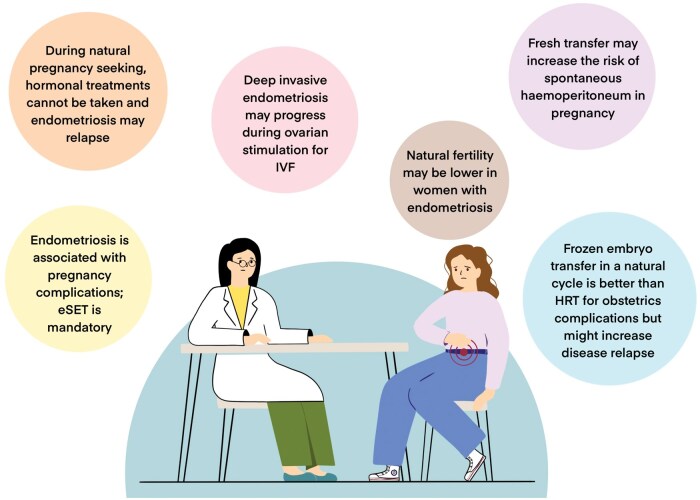
**All points to be taken in mind and discuss when considering pregnancy seeking and ART treatment in women with endometriosis.** eSET, elective single embryo transfer; HRT, hormone replacement therapy.

## Introduction

Endometriosis is a chronic, inflammatory, and recurrent disease characterized by the presence of endometrial glands, endometrial stroma, and fibrosis in ectopic locations (Vigano *et al.*, 2018; Becker *et al.*, 2022). The disease can be highly debilitating, severely impacting the quality of life by causing chronic pelvic pain in forms such as dysmenorrhea, dyspareunia, non-menstrual pain, dyschezia, and dysuria. It can also disrupt reproductive mechanisms, leading to infertility (Zondervan *et al.*, 2020; Al-Lami *et al.*, 2024).

In recent decades, the range of treatment options for endometriosis has expanded, allowing physicians to make increasingly effective use of both new and established therapies. These treatments include laparoscopic surgery, hormonal medical therapies, and ARTs, particularly IVF (Becker *et al.*, 2022).

The use of hormonal medical therapies has grown over the years, with GnRH antagonists—used with or without add-back therapies—recently entering the market (Taylor *et al.*, 2017; Giudice *et al.*, 2022; Donnez *et al.*, 2024). Conceptually, these agents are not entirely innovative in treating endometriosis. Their development is based on the old and established rationale of reducing sex steroid levels, creating a steady hormonal state, and preventing ovulation—mechanisms that are not fundamentally different from those of existing treatments like low-dose estroprogestins, progestins alone, or GnRH agonists with or without add-back therapy (Vercellini *et al.*, 2024).

The growing role of hormonal therapies reflects a rethinking of the role of surgery, which was once considered the first-line treatment for endometriosis. This shift is driven by increased awareness of the complications and long-term adverse effects of surgical intervention. For instance, removing ovarian endometriomas can severely damage ovarian reserve (Baraki *et al.*, 2024), while excising deep invasive endometriosis can compromise the integrity of nearby organs, potentially causing long-term bowel or bladder dysfunctions (Schneyer *et al.*, 2024). Currently, surgery must be considered for patients who are refractory to medical therapy and for those who cannot be prescribed these drugs; it is mandatory for cases of ureteral or colorectal endometriosis causing clinically significant stenosis (Vercellini *et al.*, 2024). Furthermore, even when a surgical intervention is decided, the disease’s tendency to return has led to the widespread use of hormonal medical therapy as a tertiary prevention measure (Somigliana *et al.*, 2014). There is growing consensus that long-term progestins or estroprogestins should be used post-surgery to prevent recurrences (Zakhari *et al.*, 2020; Petraglia *et al.*, 2024; Vercellini *et al.*, 2024). Importantly, the protective effects of these therapies last only as long as they are used. This is especially evident for endometriomas, likely due to ovulation’s key role in their pathogenesis (Vercellini *et al.*, 2010; Wattanayingcharoenchai *et al.*, 2021). Once hormonal treatment is discontinued, endometriosis can recur, exposing women to an increasing risk over time. The overall cumulative risk is directly related to the duration of time without medical treatment (Vercellini *et al.*, 2008; Zakhari *et al.*, 2021).

The role of surgery in restoring fertility is even more contentious (Vercellini *et al.*, 2009; Becker *et al.*, 2022; Ottolina *et al.*, 2022). Women undergoing surgery are exposed to the above-mentioned complications, yet the benefits for natural fertility are uncertain (Vercellini *et al.*, 2009; Ottolina *et al.*, 2022). Consequently, ART has gained increasing acceptance as the preferred approach for managing endometriosis-related infertility (Becker *et al.*, 2022).

## The dilemma of recurrences and progression in women seeking pregnancy

All established medical therapies for endometriosis hinder natural conception. GnRH agonists and antagonists, progestins, and estroprogestins all interfere with the hypothalamic–pituitary–gonadal axis and its feedback mechanisms, disrupting the delicate processes involved in ovulation. These treatments may also negatively affect sperm transit, tubal transport, and endometrial receptivity. Consequently, medical therapy is incompatible with efforts to conceive naturally. Even women undergoing ART must discontinue hormonal medical treatments during critical periods, such as ovarian stimulation cycles and frozen embryo transfers.

Notably, the growing adoption of elective single embryo transfer (eSET) has increased the number of transfer attempts required to achieve pregnancy. This, in turn, extends the time women scheduled for ART remain at risk of recurrence or progression of endometriosis. Additionally, frozen embryo transfers are increasingly performed during natural cycles rather than artificial preparation cycles, as the presence of a corpus luteum is thought to positively influence pregnancy outcomes (Busnelli *et al.*, 2022). Hormone replacement therapy for endometrial preparation is indeed associated with a higher risk of hypertensive disorders of pregnancy, pre-eclampsia, postpartum hemorrhage, and cesarean section (Busnelli *et al.*, 2022). However, during natural cycle transfers, ovulation occurs, exposing women to the risk of endometrioma’s development (Vercellini *et al.*, 2010).

Physicians and patients must carefully weigh the likelihood of achieving a natural or ART-mediated pregnancy against the risk of recurrence or progression of endometriosis during the necessary discontinuation of medical treatment. On one hand, this requires an accurate estimate of the chances of conception; on the other, an evaluation of the risk of disease recurrence or progression.

### Predicting the chance of pregnancy

Predicting natural conception in women with endometriosis remains challenging. The most reliable tool available is the Endometriosis Fertility Index (EFI), a scoring system that combines multiple factors into a score ranging from 0 to 10, which predicts natural conception following surgery. While the EFI has been repeatedly validated, it remains imprecise at the individual level (Vesali *et al.*, 2020). Moreover, the EFI is not designed to predict natural conception in women who have not undergone surgery or who had surgery long before attempting pregnancy. Some efforts have been made to validate the EFI in non-operated women, but the evidence is still weak (Tomassetti *et al.*, 2021). This limitation contrasts with the modern approach to managing endometriosis, which emphasizes minimizing surgical interventions whenever possible.

In contrast, the ability to predict the chance of pregnancy with IVF is better established and validated (Shingshetty *et al.*, 2024). Notably, in contrast to previous evidence, endometriosis *per se* does not seem to significantly influence the chance of success. Age and residual ovarian reserve are the two most significant factors influencing ART success also in women with the disease (Somigliana *et al.*, 2023). Moreover, adenomyosis, a condition commonly associated with endometriosis, may reduce IVF success (Cozzolino *et al.*, 2022). However, to date, evidence obtained with the use of modern criteria for diagnosis is contrasting and not trenchant (Neal *et al.*, 2020; Higgins *et al.*, 2021; Alson *et al.*, 2024; Cozzolino *et al.*, 2024).

### Predicting recurrence and progression

While extensive research has focused on predicting natural and ART-mediated pregnancy chance, tools for predicting individual recurrence or progression of endometriosis are less well-developed. In general, it is well known that endometriosis is a recurrent disease, with an annual recurrence rate of ∼10% (Guo, 2009). Recent studies have generally confirmed this figure. A recent meta-analysis focusing on endometriomas reported recurrence rates of 4%, 14%, 17%, and 27% at 3, 6, 12, and 24 months, respectively, after surgery (Veth *et al.*, 2024). Risk factors include age at surgery, CA125 levels, cyst size, dysmenorrhea, history of endometriosis-related surgery, pre-operative medication, and rASRM scores. However, a validated scoring system for predicting recurrence is not yet available (Jiang *et al.*, 2021).

Bendifallah *et al.* (2020) reviewed the literature on colorectal endometriosis and reported a post-surgical recurrence rate of 6–9%, which was only modestly influenced by the surgical technique employed (O’Brien *et al.*, 2023). Similarly, Alborzi *et al.* (2023) reviewed the post-surgical outcomes of women treated for ureteral endometriosis, reporting recurrence and stricture rates of 2% and 15%, respectively. These rates may underestimate the true recurrence risk, as some patients included in the studies were using medical therapies to prevent recurrence. Moreover, data on deep invasive lesions (e.g. colorectal or ureteral) are often reported as raw numbers rather than annualized rates. This lack of temporal context is particularly relevant for women attempting pregnancy, as the exposure period should ideally not exceed 1 year. Beyond this time frame, infertility may reasonably be inferred, and ART becomes necessary. Annual recurrence rates would therefore be especially informative. Overall, the recent available evidence did not change the scenario and supports an annual recurrence rate of ∼10% in the absence of medical therapy.

While recurrence rates are quite well-documented, the progression of untreated endometriotic lesions has been less thoroughly investigated. What happens to non-operated endometriotic lesions that are not managed with hormonal therapies? Bandini *et al.* recently reviewed the available literature on deep invasive lesions and identified four studies reporting progression rates under untreated conditions. Combined, these data estimate a progression rate of 21% (95% CI: 7–41%), which contrasts negatively with the 12% (95% CI: 9–16%) recurrence rate reported in women using medical therapy (Bandini *et al.*, 2024). Notably, all four studies had long follow-up periods (mean/median ranging from 22 to 65 months), suggesting that the 1-year progression risk is likely lower (again an about 10% per year).

For endometriomas, data remain sparse and has not been meta-analyzed. Knez *et al.* (2024) recently evaluated 120 endometriomas in 83 women over a median follow-up of 21 months and observed growth in 28 lesions (23%, 95% CI: 16–32%). The remaining lesions either remained stable or regressed. Previous evidence included smaller numbers of women and showed similar findings, with the rate of endometriomas’ growth varying between 8% and 24% (Ueda *et al.*, 2010; Benaglia *et al.*, 2013; Bailleux *et al.*, 2017).

Overall, women with endometriosis who are seeking natural pregnancy face a low but consistent risk of recurrence or progression. This risk is clinically acceptable only if the woman has a reasonable chance of achieving pregnancy naturally. Noteworthy, recurrences or progression here should not be merely interpreted with the reappearance or growth of sonographic images suggestive for endometriosis, they must include also subjective pain. Even if the sonographic findings remain unremarkable, the recurrence of pain may be unbearable, hampering the possibility for natural conception. Overall, a personalized and shared decision-making approach is essential in this complex clinical scenario, considering family planning wishes, surgical findings (if applicable), ultrasound findings, age, pain symptoms, previous pregnancies, semen analysis, and biomarkers of ovarian reserve. Notably, low ovarian reserve does not necessarily indicate a need to shift to IVF, as ovarian reserve is critical for IVF success but not for natural conception (Zarek *et al.*, 2015; Steiner *et al.*, 2017; Galati *et al.*, 2024).

## The impact of ovarian stimulation on endometriosis

The success of IVF is closely linked to ovarian response. A greater ovarian response to stimulation increases the number of oocytes retrieved, the availability of embryos for transfer, the cumulative chances of success, and, ultimately, the number of babies born. However, a robust ovarian response is accompanied by a significant increase in sex steroid levels, particularly estradiol. In natural cycles, estradiol levels peak at 200–300 pg/ml, whereas in cases of strong ovarian response, these levels can rise to concentrations 10 times higher (Macklon *et al.*, 2006). This inevitably raises concerns about the safety of IVF in women with endometriosis. Estrogens have a major role for endometriotic tissue ectopic implantation, survival, and growth, and for neo-angiogenesis and synthesis of inflammatory mediators as well. Pathological studies have clearly documented widespread estrogen receptor (ER) expression in endometriotic lesions, particularly in cases of deep endometriosis. An overexpression of ERβ and a downregulation of ERα have also been observed in endometriosis (Clemenza *et al.*, 2022; Vannuccini *et al.*, 2022).

Understanding the potential risks of IVF in terms of disease recurrence and progression is essential given the current emphasis on ART to treat endometriosis-related infertility. It is vital to establish whether this approach poses additional risks to women, as this information is crucial for effective counseling and shared decision-making. Available evidence is generally reassuring (Somigliana *et al.*, 2019; Benaglia *et al.*, 2021a). A systematic review of 16 studies revealed that ovarian stimulation for IVF has minimal impact on the disease (Somigliana *et al.*, 2019). IVF does not exacerbate pain symptoms, increase recurrence risk, or significantly affect endometrioma size. However, limited evidence suggests that deep endometriosis may progress with ovarian stimulation (Somigliana *et al.*, 2019; Benaglia *et al.*, 2021a). This concern was supported by five case reports (in total 12 cases) of progression (Renier *et al.*, 1995; Govaerts *et al.*, 1998; Anaf *et al.*, 2000; Jun and Lathi, 2007; Halvorson *et al.*, 2012). This issue is particularly critical because deep endometriosis can lead to serious complications, such as obstructive uropathy or bowel occlusion.

To further address this concern, we have updated our previous systematic review to include evidence published from January 2018 to December 2024 (the previous review included studies up to January 2018), and focusing specifically on deep endometriosis. Five new studies were identified. These included three additional case reports documenting progression (five cases in total: three involving thoracic endometriosis, one with colon obstruction, and one with ureteral obstruction) (Seyer-Hansen *et al.*, 2018; Thoreau *et al.*, 2018; Pellerin *et al.*, 2020). More importantly, two cohort studies provided more robust evidence (Seyer-Hansen *et al.*, 2018; Berlanda *et al.*, 2019). In the first study, Seyer-Hansen *et al.* examined 77 women with non-operated bowel deep endometriosis who underwent IVF. Severe worsening of symptoms was observed in 10 women (13%, 95% CI: 7–22%). Among these, two experienced colon occlusion, six had sub-occlusion, and two reported worsening pain. All 10 women required a surgical intervention. In contrast, data from Berlanda *et al.* (2019) painted a less alarming picture. This study reviewed 60 women with deep endometriosis who had failed IVF and compared lesion dimensions before and after treatment. The mean diameter of endometriotic nodules before and after IVF was similar (19 ± 6 and 18 ± 7 mm, respectively). Pain symptoms remained stable across the cohort, and no new nodules were detected. Only one woman (2%, 95% CI: 0.1–8%) experienced a significant complication. She had a transient ureteral stenosis, which resolved with expectant management without requiring surgery. The reasons behind the inconsistency between these two studies are difficult to unravel. One possible explanation is that women in the study by Seyer-Hansen *et al.* had more severe endometriosis, as it exclusively included cases of deep bowel endometriosis, whereas the study by Berlanda and collaborators included deep endometriosis at any location. Further research is needed to clarify these discrepancies and better understand the risks associated with IVF in women with deep endometriosis.

Finally, the impact of ovarian stimulation on adenomyosis is a neglected but potentially important issue. Unfortunately, we failed to identify informative studies specifically investigating this topic. The evaluation of the transformation of adenomyosis in pregnancy is demanding (myometrium also rapidly expands and changes) and this may explain the lack of evidence.

## Endometriosis during pregnancy

Concerns about the impact of ovarian stimulation on endometriosis growth should not be limited to the time of IVF treatment. It is also essential to consider the potential additive or amplifying effects of a pregnancy achieved through a fresh ART cycle, i.e. immediately following ovarian stimulation. The combined influences of multiple corpora lutea and the ability of hCG to sustain their activity for several weeks may exacerbate endometriosis. In other words, it is necessary to clarify whether pregnancy outcomes differ between women with endometriosis who conceive naturally and those who require ART.

The effect of hormonal, biochemical, and metabolic changes associated with pregnancy on endometriotic lesions has been extensively studied in recent years, with most evidence focusing on ovarian endometriomas. Research has consistently shown that these lesions can undergo decidualization early in pregnancy, adopting a sonographic appearance that can mimic malignancy (Bean *et al.*, 2022, 2023; Filippi *et al.*, 2022). The impact of pregnancy on deep invasive endometriosis has been harder to study and has received less attention, but emerging evidence offers some insights (Bean *et al.*, 2022, 2023; Filippi *et al.*, 2022; Zajicek *et al.*, 2024). Deep nodules can also decidualize, becoming hyperechoic with moderate to high vascularity on color Doppler imaging. However, this transformation tends to occur later in pregnancy. Recent findings suggest that the frequency of decidualization in endometriomas and deep nodules is higher than previously thought, occurring in up to 50% of cases (Filippi *et al.*, 2022; Bean *et al.*, 2023; Zajicek *et al.*, 2024).

Unfortunately, there is currently insufficient evidence to determine whether the frequency or severity of decidualization is influenced by ART. The presence of multiple corpora lutea and elevated peripheral sex steroid levels in ART cycles could potentially amplify this effect. Although a trend in this direction has been noted, the available evidence is insufficient for definitive conclusions (Filippi *et al.*, 2022). Moreover, what are the clinical consequences of this ectopic decidualization?

Endometriosis has been linked to several obstetric complications. Although the evidence is not entirely consistent, studies suggest a modest increase in risks for gestational hypertension, gestational diabetes, preeclampsia, preterm premature rupture of membranes, preterm delivery, cesarean section rates, antepartum bleeding, postpartum hemorrhage, and adverse neonatal outcomes (Salmeri *et al.*, 2023; Vercellini *et al.*, 2023; Busnelli *et al.*, 2024; Enzelsberger *et al.*, 2024). The most pronounced association, however, is with placenta previa, where women with severe endometriosis face a 5- to 10-fold increased risk (Vercellini *et al.*, 2023). However, a direct influence of ectopic lesions on these obstetric disorders, including placenta previa, is unlikely. Instead, these effects are likely mediated by the strong association between endometriosis and adenomyosis (Vercellini *et al.*, 2023). Consequently, concerns about obstetric complications should not heavily influence the decision to pursue natural conception versus ART.

On the other hand, women with endometriosis face a higher risk of spontaneous hemoperitoneum during pregnancy, a rare but severe complication associated with a significant risk of fetal loss and even maternal death (Brosens *et al.*, 2016; Lier *et al.*, 2017; Benaglia *et al.*, 2021b; Schreurs *et al.*, 2023). This severe complication is extremely rare (<1%), develops progressively due to persistent bleeding from venous vessels, and sometimes without clear, pathognomonic symptoms, thus potentially hampering a prompt diagnosis. Women must be informed about it. The risk appears to be higher in pregnancies achieved through ART and in the presence of deep invasive endometriosis (Brosens *et al.*, 2016; Lier *et al.*, 2017; Benaglia *et al.*, 2021b). The decidualization of deep invasive lesions may contribute to this risk. While firm conclusions are difficult to draw due to the rarity of the condition, it may be prudent to consider avoiding fresh embryo transfers in women with unoperated deep invasive endometriosis to minimize the impact of multiple corpora lutea. However, a firm recommendation for freeze all in women with deep invasive endometriosis cannot be drawn. Our considerations are speculative, robust data are not available.

Finally, it is worth emphasizing that other potential complications of ectopic decidualization, such as intestinal or ureteral obstruction, intestinal perforation or fistula, can occur during pregnancy. Their frequency is presumably very rare and only supported by case reports (Leone Roberti Maggiore *et al.*, 2017). However, a detrimental additional effect of ART is not demonstrated here.

## Conclusions

The choice between attempting natural conception and proceeding directly to IVF is complex and requires careful consideration of several critical, yet often poorly defined, factors. These include evaluating the likelihood of achieving a natural pregnancy, the probability of success with ART, the effect on pain symptoms, the risk of endometriosis progression associated with repeated natural cycles or ART, and the potential impact on pregnancy complications. In general, pursuing natural pregnancy may expose women to a slightly higher risk of endometriosis progression or recurrence compared to ART, but this risk is relatively low—∼10% over the typical 1-year timeframe. On the other hand, ART is more costly and carries its own specific risks. The decision-making process cannot be rigid or one-size-fits-all. Instead, management should be personalized and tailored to the individual woman’s preferences and clinical condition. Factors such as her medical history, sonographic findings, semen analysis results, and the presence of pain symptoms must all be carefully considered. Tailored approaches might include adjusting the duration of natural conception attempts (e.g. shortening to 6 months or extending beyond 12 months), closely monitoring the condition during attempts at natural conception, and initiating medical therapy promptly in cases of endometriosis recurrence or progression.

In ART cycles, evidence is too weak for strong recommendations. However, some speculative suggestions may be drawn. Strategies such as mandatory eSET, transfer at blastocyst stage to lower the number of transfers, avoiding fresh embryo transfers to avoid a pregnancy in a hyper-stimulated hormonal context that could facilitate decidualization, or using artificial endometrial preparation to avoid ovulation may be considered. For the two latest interventions, one must balance the decision with the drawbacks of a policy of systematic freeze all and the potential beneficial effects of embryo transfer in a natural cycle on pregnancy complications (lowering the risk of hypertensive disorders of pregnancy, preeclampsia, postpartum hemorrhage, and cesarean section). Even if still debated because of the risk of discarding valuable embryos, the use of preimplantation genetic testing for aneuploidy (PGT-A) might be considered here because it can reduce the number of transfers and shorten exposure to ovulations (Viville and Aboulghar, 2025). Finally, when planning for ovarian stimulation one could consider a progesterone-primed ovarian stimulation treatment. In this protocol, women receive progesterone or a progestin to prevent ovulation all along the stimulation and this could theoretically be beneficial for endometriosis. However, this protocol is incompatible with fresh embryo transfer. Even if the benefits are theoretical, it seems wise to consider this approach when fresh transfer is excluded *a priori*. Noteworthy, in most extreme scenarios when risks are deemed excessive, one could opt straight for oocytes donation or even refraining from conceiving (Vercellini *et al.*, 2018).

In conclusion, the impact of discontinuing medical therapy to pursue natural conception or ART is an under-researched area with limited and indirect evidence. Nevertheless, this is a critical issue for women with endometriosis and warrants greater attention in the future. In the meantime, a shared decision-making approach is essential, incorporating all aspects of this multifaceted clinical issue to ensure that decisions align with the individual’s preferences and condition.

## Data Availability

Not applicable.
